# Bruch's Membrane Opening Minimum Rim Width Measurement with SD-OCT: A Method to Correct for the Opening Size of Bruch's Membrane

**DOI:** 10.1155/2017/8963267

**Published:** 2017-10-02

**Authors:** Robert Kromer, Martin Stephan Spitzer

**Affiliations:** Department of Ophthalmology, University Hospital Hamburg-Eppendorf, Hamburg, Germany

## Abstract

A precise evaluation of the retinal nerve fiber layer thickness (RNFLT) is key for diagnosing and monitoring glaucoma. The Bruch's membrane opening minimum rim width (BMO-MRW) has been proposed as a reproducible assessment of the optic nerve. The BMO-MRW measures the minimum distance from the BMO to the internal limiting membrane. We propose an approach to correct the BMO-MRW using the BMO size for increased accuracy in interindividual comparisons in future studies. Eighty-one healthy patients received SPECTRALIS spectral domain optical coherence tomography measurements for the peripapillary RNFLT and BMO-MRW. We calculated a BMO size-corrected BMO-MRW using the mean BMO size of our cohort. BMO size was defined using the manufacturer-provided BMO area and manually measured BMO perimeter. We observed that the BMO-MRW correlated highly with the perimeter (*r* = −0.553, *p* < 0.0001) and the area of the BMO (*r* = −0.546, *p* < 0.0001). Using these parameters, we provided a corrected BMO size-adjusted BMO-MRW which was better correlated with the RNFLT compared to the noncorrected one (*z* = −3.3495, *p* = 0.0004). We demonstrated the dependency of the BMO-MRW on ONH size. Furthermore, we showed the superiority of the corrected BMO-MRW using either the manually measured optic nerve head perimeter or the automatically provided ONH for future studies.

## 1. Introduction

Glaucoma is one of the main causes of impaired vision worldwide [1]. An early and sufficient reduction of intraocular pressure significantly improve patient outcomes [2, 3]. Therefore, it is important to objectively and reliably detect the disease and monitor structural defects throughout. Patients develop characteristic changes, such as retinal nerve fiber layer thickness (RNFLT) loss, neuroretinal rim thinning, or increasing excavation cup depth prior to experiencing visual field defects [4, 5]. It has been shown that a precise evaluation of the retinal nerve fiber layer (RNFL) is key for diagnosing and monitoring glaucoma. The RNFL is measured using optical coherence tomography (OCT), a noninvasive technology that yields high-resolution, cross-sectional images of biological tissue [6]. The evaluation of the peripapillary RNFL is widely used to describe objective and reliable information on glaucomatous optic nerve damage [7–11]. Recently, a new parameter for evaluation has been proposed for reproducible assessments of the optic nerve: the Bruch's membrane opening minimum rim width (BMO-MRW). The BMO-MRW measures the minimum distance from the inner opening of the BMO to the internal limiting membrane (ILM). It uses stable borders and offers a more accurate geometric assessment of neuroretinal rim tissue [12–16]. The BMO seems to remain stable over time and can therefore be used as a reference point [17]. Thus, the BMO-MRW was demonstrated to have a stronger association with visual field sensitivity than other parameters [18, 19] and comparable accuracy for the discrimination of pre- and perimetric glaucomatous eyes using the RNFLT [16].

The optic nerve head (ONH) size is not consistent among individuals and shows an interindividual variability in the area ranging from about 0.80 mm^2^ to almost 6.00 mm^2^ in a normal Caucasian population [5]. It has been hypothesized that the ONH size is potentially correlated with glaucoma susceptibility. Currently, the prevailing opinion and evidence are that the disc size has barely any or no effect on glaucoma as different possible effects compensate for each other and the peripapillary RNFLT is independent of disc size [5, 20]. ONH size is important, as eyes with larger ONHs have less nerve fiber crowding per square millimeter of the disc area [21].

**Figure 1:**
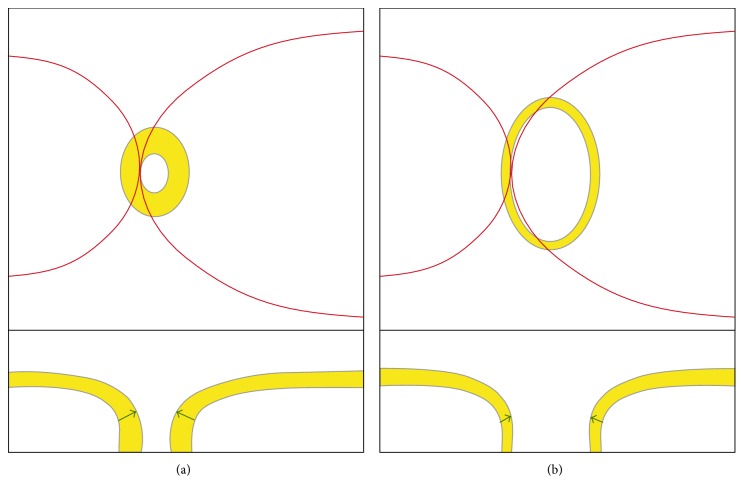
A schematic illustration of two different ONH sizes: (a) small and (b) large. The upper images depict fundus images with red main vessels and the optic nerves (the neuroretinal rim area is marked in yellow and the excavation is marked in white inside the ONH), while the lower images feature horizontal cross-sectional OCT images through the middle of the optic nerves (with a yellow RNFL). The neuroretinal rim areas, and therefore the total number of axons, are equal in both (a) and (b). However, the BMO-MRW (indicated by green arrows in the lower images) is reduced in image (b). The hypothesis is that the BMO-MRW is influenced by ONH size and requires correction for use in valid comparisons within a cohort.

Furthermore, with larger ONH sizes, the retinal nerves are spread over an increased perimeter, and therefore, the measured BMO-MRW seems to be diminished while still possessing an equivalent number of individual nerves, as demonstrated in Figure 1. The dependency of the BMO-MRW on the ONH size is well known and has been shown in studies [15, 22]. The manufacturers' normative BMO-MRW values are adjusted for age and BMO size. However, several studies did not correct for optic disc size or BMO size when performing interindividual comparisons [18, 19, 22–24].

BMO size can be described by its area or perimeter. In the literature, the BMO area has been the only parameter used to adjust the BMO-MRW. In this study, we propose an approach to correct the BMO-MRW by BMO size to increase accuracy in interindividual comparisons in future studies and evaluate whether the BMO area or perimeter has greater accuracy in describing its size.

## 2. Methods

We included healthy patients recruited retrospectively from the University Ophthalmology Center Hamburg-Eppendorf, Hamburg, Germany. The Ethics Committee of the Medical Association of Hamburg, Germany, ruled that no patient consent was required as all evaluations were performed retrospectively and anonymously.

Each participant's history and medical records were carefully reviewed for retinal diseases. Only participants satisfying the inclusion criteria and not meeting any exclusion criterion were included. The ophthalmic inclusion criteria were as follows: (i) a best-corrected visual acuity of 0.1 logMAR or better; (ii) a spherical equivalent within ± 3.0 diopters; and (iii) a cylindrical correction within ± 1.0 D. The exclusion criteria were as follows: (i) intense alcohol abuse; (ii) a body mass index > 30 kg/m^2^; (iii) an intraocular pressure ≥ 21 mmHg; (iv) known ophthalmic diseases; and (v) congenital abnormalities of the optic nerve.

The medical records had to include the following ophthalmic examinations at minimum: (i) subjective refractometry; (ii) slit lamp-assisted biomicroscopy of the anterior segment; (iii) ophthalmoscopy; (iv) visual field testing (Humphrey Visual Field Analyzer 30-2 (76 points over the central 30° of the visual field); Humphrey, San Leandro, CA, USA); (v) Goldmann applanation tonometry; and (iv) spectral-domain OCT (SD-OCT) measurement (SPECTRALIS; version 6.5.2.0; Heidelberg Engineering, Heidelberg, Germany).

The SPECTRALIS SD-OCT was performed with every scan accompanying confocal scanning laser ophthalmoscopy (cSLO) images of the fundus. This methodology obtained noncontact frames of the retina at a high resolution. A superluminescent diode was employed to emit a light beam at a wavelength of 870 nm. The SD-OCT system can receive up to 40,000 A-scans per second with a depth resolution of 7 *μ*m and a transverse resolution of 14 *μ*m. An additional feature, the automatic real-time averaging mode (ART), resulted in the achievement of even higher quality. In this study, high-quality SD-OCT scans with an average of 100 frames were used to provide images with low noise. We used the proprietary anatomic positioning system (APS) method, which is based on manually located points in the eye using two fixed structural landmarks: the center of the fovea and the center of the BMO [22]. These landmarks are defined during the initial APS scan. Each participant underwent star-pattern acquisition of the ONH consisting of 24 scans for the BMO-MRW and three high-resolution peripapillary SD-OCT scans with different diameters (3.5 mm, 4.1 mm, and 4.7 mm). Images that did not meet the following quality criteria were excluded: absence of a scan, algorithm failures, and consistency of the grayscale saturation of each RNFL with the retinal pigment epithelium showing maximal shading. Furthermore, OCT scans had to satisfy consensus criteria for retinal OCT quality assessment (OSCAR-IB) to improve the comparability and quality management of the OCT images [25].

**Figure 2:**
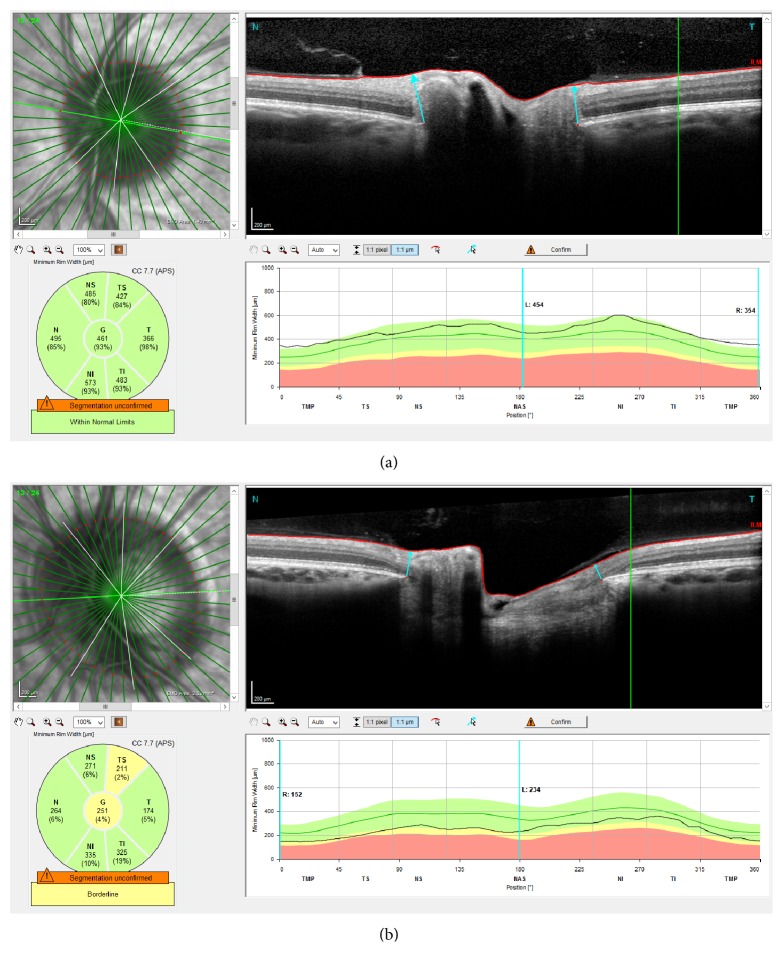
A sample demonstration of two representative SPECTRALIS SD-OCT BMO-MRW examinations. The upper left image shows the cSLO image of the ONH with red-marked borders of the perimeter as established by the BMO and green lines indicating the acquired SD-OCT scans. The right image shows one SD-OCT scan with a marked BMO (small red dot) and the BMO-MRW (cyan arrow). The lower left schematic shows the mean BMO-MRW for the individual sectors and the lower right schematic shows the distribution of the BMO-MRW over 360° of the ONH. The black line indicates the actual measurement while the colors (green: within normal borders, yellow: borderline, red: outside normal borders) represent the underlying normative values. Image (a) shows a small ONH with an area of 1.40 mm^2^ and a manually measured perimeter of 4244.2 *μ*m. The BMO-MRW is in the upper range of the manufacturer-provided normative values. Image (b) shows a large ONH with an area of 2.33 mm^2^ and a perimeter of 5454.9 *μ*m; the BMO-MRW is in the lower range of the normative values.

Our hypothesis was that the BMO-MRW is influenced by ONH size. This is exemplified in Figure 2, in which Figure 2(a) shows a small ONH with a BMO-MRW in the upper range of the manufacturer-provided normative values and Figure 2(b) shows a large optic nerve and a BMO-MRW in the lower range of the normative values. We thus calculated a BMO size-corrected BMO-MRW (cBMO-MRW) using the following formula:

(1) $$\begin{array}{c} cBMO‐MRW=\frac{ONH\;size}{mean\;ONH\;size}\cdot BMO‐MRW. \end{array}$$

BMO size can be described using the area and perimeter. The SPECTRALIS software calculates the BMO area; however, it does not provide the BMO perimeter based on the BMO-defined size. Therefore, the BMO perimeter had to be measured manually. Initially, the cSLO images with marked BMO positions (as red points) were exported from the Heidelberg Eye Explorer as a standardized screenshot. All images were processed by one examiner (R. K.). Next, the screenshots were imported into open-source software, ImageJ for Windows, v. 1.80 (http://imagej.nih.gov/ij/; provided in the public domain by the National Institutes of Health, Bethesda, MD, USA). ImageJ was utilized to precisely measure the area and perimeter in pixels following the positioned BMO points in the cSLO scan (comparison in Figure 2()). The BMO-defined ONH area was also provided by the SPECTRALIS software in mm^2^. A comparison between the provided BMO area (mm^2^) and the manually measured area in pixels was performed, and we were able to determine a scale and converted the pixels along the perimeter into micrometers. We used the manually measured perimeter as well as the software-provided area to correct the BMO-MRW and compared the results from both approaches. The mean BMO area and perimeter were defined as the mean values from our sample.

The statistical analysis was performed with commercially available software packages (Prism 7 for Mac OSX; GraphPad Software Inc., La Jolla, USA; Version 7.0a). The means and standard deviations are presented, and *p* values were corrected for multiple comparisons according to the Bonferroni method. Paired parametric *t*-tests were applied and *p* values were two tailed, with a *p* value < 0.05 considered to indicate statistical significance. The correlation was performed using Pearson correlation calculations, as the values sampled from the populations followed an approximate Gaussian distribution. The correlation coefficient was indicated by the *r* value. Plotted correlations and linear regressions are included. The statistical comparison of correlations was carried out using an open-source software package (R for Mac OSX; R Core Team, GNU GPL; Version 3.3.2) and the Cocor software package ([26]; Version 1.1–3). The tests proposed by Meng et al. [27] were employed to compare correlation coefficients and confidence intervals (alpha level = 0.05, confidence level = 0.95). The null hypothesis was rejected when the confidence interval included zero. Only one eye (the right) of each participant was selected for the statistical analyses (phenotype).

## 3. Results

**Figure 3:**
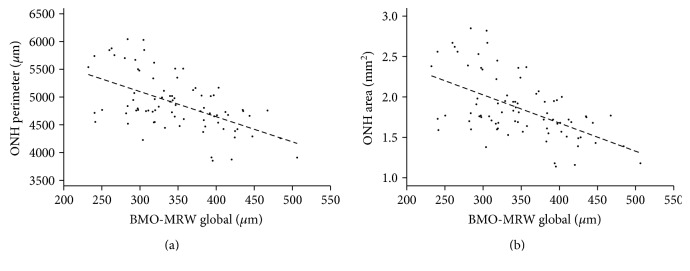
Correlations with linear regressions of the mean global BMO-MRW and perimeter ((a) *r* = −0.553, *p* < 0.0001) or area ((b) *r* = −0.546, *p* < 0.0001) of the BMO are shown.

A total of 81 right eyes in 81 healthy patients were included (mean age: 24.8 years; standard deviation: 3.4 years; 50 women). The manufacturer-provided software measured the mean global (G)-RNFLT and separated it into six sectors (temporal (T), temporal-superior (TS), temporal-inferior (TI), nasal (N), nasal-superior (NS), and nasal-inferior (NI)) for each of the three different diameters (3.5 mm, 4.1 mm, and 4.7 mm) from the geometric center of the ONH. Furthermore, the BMO-MRW was provided both globally and for the same six aforementioned sectors. There was a significant correlation between the G-BMO-MRW and all sectors and with the perimeter (*G*: *r* = −0.553, *p* < 0.0001; compared with that in Figure 3(a)) and area (*G*: *r* = −0.546, *p* < 0.0001; compared with that in Figure 3(b)) of the BMO; individual values are provided in Table 1.

The BMO-MRW was corrected using the perimeter (cpBMO-MRW) and the area (caBMO-MRW) as described earlier. The BMO-MRW was not different from the cpBMO-MRW (mean of the differences: −3.40 ± 35.21 *μ*m; *p* = 0.387) or the caBMO-MRW (mean of the differences: −7.13 ± 72.23 *μ*m; *p* = 0.377). The corrected BMO-MRW values also showed no significant difference (mean of the differences: −3.73 ± 37.75 *μ*m; *p* = 0.376).

There was no significant correlation between the BMO perimeter and RNFLT or between the BMO area and RNFLT (*p* > 0.05).

**Figure 4:**
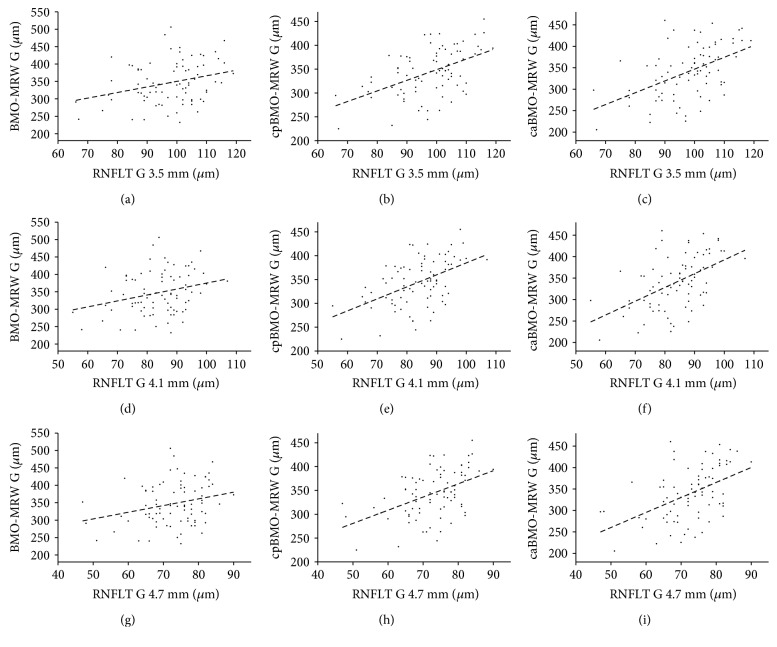
Correlations with linear regressions are shown of the mean 3.5 mm diameter global RNFLT with the mean global BMO-MRW (*r* = 0.292; *p* = 0.009 (a)), the mean global perimeter-based corrected BMO-MRW (*r* = 0.501; *p* < 0.0001 (b)), the mean global area-based corrected BMO-MRW (*r* = 0.502; *p* < 0.0001 (c)); the correlation of the mean 4.1 mm diameter global RNFLT with the mean global BMO-MRW (*r* = 0.272; *p* = 0.014 (d)), the mean global perimeter-based corrected BMO-MRW (*r* = 0.496; *p* < 0.0001 (e)), the mean global area-based corrected BMO-MRW (*r* = 0.514; *p* < 0.0001 (f)), the correlation of the mean 4.7 mm diameter global RNFLT with the mean global BMO-MRW (*r* = 0.265; *p* = 0.017 (g)), the mean global perimeter-based corrected BMO-MRW (*r* = 0.473; *p* < 0.0001 (h)), and the mean global area-based corrected BMO-MRW (*r* = 0.486; *p* < 0.0001 (i)).

The correlations between the mean 3.5 mm diameter G-RNFLT, the G-BMO-MRW, and the corrected variants were significant for all RNFL diameters (RNFLT versus BMO-MRW: *r* = 0.292, *p* = 0.009; RNFLT versus BMO-MRW: *r* = 0.501, *p* < 0.0001; RNFLT versus BMO-MRW: *r* = 0.502, *p* < 0.0001). There was no significant correlation between the mean RNFLT for all diameters, for the T sector and the BMO-MRW, or for the corrected BMO-MRWs. Furthermore, the uncorrected BMO-MRW did not significantly correlate with the 4.1 mm diameter RNFLT in the TS sector or the 4.7 mm diameter RNFLTs in the TS and N sectors. Individual values with results for the remaining diameters are featured in Table 2. The correlations for *G* values are plotted via linear regressions in Figures 4(a), 4(b), 4(c), 4(d), 4(e), 4(f), 4(g), 4(h), and 4(i).

The comparisons of correlation coefficients and their confidence intervals for the 3.5 mm G-RNFLT versus the BMO-MRW and the 3.5 mm G-RNFLT versus the cpBMO-MRW exhibited a significant difference (*z* = −3.3495, *p* = 0.0004; 95% confidence interval: −0.3961 to −0.1037), demonstrating the superiority of the cpBMO-MRW over the BMO-MRW in terms of the correlation with the RNFLT. The comparison of the correlation coefficients for the 3.5 mm G-RNFLT versus the BMO-MRW and the 3.5 mm G-RNFLT versus the caBMO-MRW did indeed demonstrate a significant difference (*z* = −1.7669, *p* = 0.0386); however, this was not the case for the confidence interval (95% confidence interval: −0.5299 to 0.0274). This demonstrated that there is a high probability that the caBMO-MRW is superior to the BMO-MRW in terms of its correlation with the RNFLT. We further compared the different correcting approaches in terms of their correlation coefficients with the RNFLT. The correlation coefficients and their confidence intervals comparing the 3.5 mm G-RNFLT versus the cpBMO-MRW and the 3.5 mm G-RNFLT versus the caBMO-MRW showed no significant difference (*z* = −0.0158, *p* = 0.9874; 95% confidence interval: −0.1672 to 0.1645).

## 4. Discussion

We observed that the BMO-MRW correlated highly with the perimeter and the area of the BMO. Using these two parameters, we provided a corrected BMO and size-adjusted BMO-MRW. We were able to demonstrate that the adjusted BMO-MRW was better correlated with the circular peripapillary RNFLT compared with the nonadjusted value. Furthermore, it seemed that the perimeter-adjusted BMO-MRW had a slightly superior correlation than the circular peripapillary RNFLT versus the area-adjusted BMO-MRW, though there was no significant difference (*p* = 0.9874).

The BMO-MRW has been proposed as a new parameter for ONH evaluation in glaucoma and is being actively researched. Studies claimed that the BMO-MRW provided high diagnostic accuracy in glaucoma [19, 22, 23, 28] and a stronger relationship with conventional rim parameters [18, 19]. Chauhan et al. [22] stated that the BMO-MRW offered better performance for discriminating glaucoma than OCT measurements of the RNFLT. Vianna et al. [23] described significantly different BMO-MRW values between healthy patients and patients with glaucoma in terms of the BMO-MRW and other findings. However, both studies showed significantly larger BMO areas in patients with glaucoma; hence, the described differences might be caused by different BMO areas. Pollet-Villard et al. [19] found a significantly stronger structure-function relationship with the BMO-MRW than with other ONH SD-OCT parameters. However, their study included different BMO areas for the selected study groups: patients with glaucoma, patients suspected of having glaucoma, and healthy patients. Several studies did not report on or include the BMO area [18, 24, 28]. Gmeiner et al. [16] and Enders et al. [29] used the BMO area to define inclusion and exclusion criteria, thus resulting in more homogenous groups. Both studies were not able to reproduce the superiority of the BMO-MRW over the RNFLT described beforehand. At very high levels of specificity, which are needed for clinical practice because of the low prevalence of the disease, Gmeiner et al. [16] even found a significantly greater diagnostic performance for the RNFLT than for the BMO-MRW.

Our results demonstrate the high dependency of the BMO-MRW on the BMO size. The BMO-MRW in its uncorrected form appears to be a joint parameter combining the equivalence of the RNFL with the influence of ONH size, shape, and further configuration information. Even though the BMO-MRW demonstrated a certain degree of enhancement over other approaches, it is disputable whether the RNFL equivalent or one of the other influential factors led to this. Following the opinion that the disc size has barely any or no effect on glaucoma [5, 20], it seems reasonable to correct the BMO-MRW using the disc size to emphasize other factors. Furthermore, we were able to show that the manually measured perimeter might have minor superiority compared with the automatically provided BMO area.

A potential alternative to a BMO size-corrected BMO-MRW might be the BMO minimum rim area. It describes the minimum surface spanned between the BMO and ILM calculated as a trapezoid and might equalize disc size dependency [30, 31].

Some potential limitations of the study are worth mentioning. First, we only corrected for BMO size but not for any other potential influences such as shape. Second, patients with retinal abnormalities or diseases were not included. The strengths of our study were as follows: (i) a healthy young patient group without retinal diseases; (ii) a manual measurement of the BMO perimeter; and (iii) the use of a standardized manufacturer-proposed acquisition technique.

In summary, we demonstrated the dependency of the BMO-MRW on BMO size as shown in previous studies. The BMO perimeter seems to have a slight superiority over the BMO area and might be preferred when the BMO size-adjusted BMO-MRW is used for interindividual comparisons in future studies.

## Figures and Tables

**Table 1:** Correlations of the BMO-MRW with the perimeter and area in the columns are shown.

BMO-MRW	BMO perimeter	BMO area
G	−0.553^∗∗∗^	−0.546^∗∗∗^
T	−0.419^∗∗∗^	−0.410^∗∗∗^
TS	−0.533^∗∗∗^	−0.517^∗∗∗^
TI	−0.424^∗∗∗^	−0.416^∗∗∗^
N	−0.539^∗∗∗^	−0.536^∗∗∗^
NS	−0.540^∗∗∗^	−0.530^∗∗∗^
NI	−0.481^∗∗∗^	−0.478^∗∗∗^

The correlation coefficient, *r*, is shown with asterisks demonstrating significance (^∗∗∗^: *p* ≤ 0.001). The results are divided into global (G) and six sectors (temporal (T), temporal-superior (TS), temporal-inferior (TI), nasal (N), nasal-superior (NS), and nasal-inferior (NI)).

**Table 2:** Correlations of the mean RNFLT at different diameters with the BMO-MRW, the perimeter-corrected BMO-MRW (cpBMO-MRW), and the area-corrected BMO-MRW (caBMO-MRW) are shown.

RNFLT	BMO-MRW	cpBMO-MRW	caBMO-MRW
3.5 mm G	0.292^∗∗^	0.501^∗∗∗^	0.502^∗∗∗^
3.5 mm T	0.108	0.182	0.191
3.5 mm TS	0.225^∗^	0.396^∗∗∗^	0.431^∗∗∗^
3.5 mm TI	0.230^∗^	0.338^∗∗^	0.304^∗^
3.5 mm N	0.251^∗^	0.395^∗∗∗^	0.435^∗∗∗^
3.5 mm NS	0.257^∗^	0.427^∗∗∗^	0.455^∗∗∗^
3.5 mm NI	0.305^∗∗^	0.370^∗∗∗^	0.319^∗∗^
4.1 mm G	0.272^∗^	0.496^∗∗∗^	0.514^∗∗∗^
4.1 mm T	0.121	0.203	0.211
4.1 mm TS	0.179	0.345^∗∗^	0.405^∗∗∗^
4.1 mm TI	0.262^∗^	0.391^∗∗∗^	0.360^∗∗∗^
4.1 mm N	0.229^∗^	0.374^∗∗∗^	0.421^∗∗∗^
4.1 mm NS	0.230^∗^	0.340^∗∗∗^	0.433^∗∗∗^
4.1 mm NI	0.231^∗^	0.300^∗∗^	0.270^∗^
4.7 mm G	0.265^∗^	0.473^∗∗∗^	0.486^∗∗∗^
4.7 mm T	0.115	0.159	0.144
4.7 mm TS	0.159	0.341^∗∗∗^	0.421^∗∗∗^
4.7 mm TI	0.297^∗∗^	0.425^∗∗^	0.390^∗∗∗^
4.7 mm N	0.181	0.335^∗∗∗^	0.397^∗∗∗^
4.7 mm NS	0.237^∗^	0.404^∗∗^	0.431^∗∗∗^
4.7 mm NI	0.229^∗^	0.296^∗∗^	0.261^∗^

The correlation coefficient, *r*, is shown with asterisks demonstrating significance (^∗^*p* ≤ 0.5, ^∗∗^*p* ≤ 0.01, ^∗∗∗^*p* ≤ 0.001). The results are divided into global (G) and six sectors (temporal (T), temporal-superior (TS), temporal-inferior (TI), nasal (N), nasal-superior (NS), and nasal-inferior (NI)).

## Abbreviations

G: Global
T: Temporal
TS: Temporal-superior
TI: Temporal-inferior
N: Nasal
NS: Nasal-superior
NI: Nasal-inferior
RNFL: Retinal nerve fiber layer
RNFLT: Retinal nerve fiber layer thickness
OCT: Optical coherence tomography
SD-OCT: Spectral-domain optical coherence tomography
cSLO: Confocal scanning laser ophthalmoscopy
APS: Anatomic positioning system
ONH: Optic nerve head
BMO-MRW: Bruch's membrane opening minimum rim width
cBMO-MRW: Bruch's membrane opening size-corrected BMO-MRW
cpBMO-MRW: Bruch's membrane opening perimeter-corrected BMO-MRW
caBMO-MRW: Bruch's membrane opening area-corrected BMO-MRW.
